# The efficacy and safety of neoadjuvant and adjuvant chemo(radio)therapy combined with surgery in patients with locally advanced rectal cancer harboring defective mismatch repair system: a large-scale multicenter propensity score analysis

**DOI:** 10.3389/fimmu.2025.1626438

**Published:** 2025-07-07

**Authors:** Huan-Huan Wang, Yuan-Yuan Yan, Hong-Yu Zeng, Yong Wang, Ke-Min Ni, Xin-Ru Yu, Jin-Ming Shi, Hong-Li Li, Jun-Feng Wang, Zhi-Yong Yuan, Qing-Lian Wen, Nicholas G. Zaorsky, Chun-Ze Zhang, Feng-Lin Zang, Mao-Bin Meng

**Affiliations:** ^1^ Department of Radiation Oncology and CyberKnife Center, Tianjin Medical University Cancer Institute and Hospital, Key Laboratory of Cancer Prevention and Therapy, Tianjin’s Clinical Research Center for Cancer, National Clinical Research Center for Cancer, Tianjin, China; ^2^ Department of Pathology, Tianjin Medical University Cancer Institute and Hospital, Key Laboratory of Cancer Prevention and Therapy, Tianjin’s Clinical Research Center for Cancer, National Clinical Research Center for Cancer, Tianjin, China; ^3^ Department of Colorectal Surgery, Tianjin Union Medical Center, Nankai University, Tianjin, China; ^4^ Department of Epidemiology and Biostatistics, Tianjin Medical University Cancer Institute and Hospital, Key Laboratory of Molecular Cancer Epidemiology, Key Laboratory of Prevention and Control of Human Major Diseases, Ministry of Education, Tianjin’s Clinical Research Center for Cancer, National Clinical Research Center for Cancer, Tianjin, China; ^5^ Department of GI Medical Oncology, Tianjin Medical University Cancer Institute and Hospital, Key Laboratory of Cancer Prevention and Therapy, Tianjin’s Clinical Research Center for Cancer, National Clinical Research Center for Cancer, Tianjin, China; ^6^ Department of Colorectal Oncology, Tianjin Medical University Cancer Institute and Hospital, Key Laboratory of Cancer Prevention and Therapy, Tianjin’s Clinical Research Center for Cancer, National Clinical Research Center for Cancer, Tianjin, China; ^7^ Department of Radiation Oncology, West China Hospital, West China School of Medicine, Sichuan University, Chengdu, China; ^8^ Department of Radiation Oncology, University Hospital Seidman Cancer Center, Case Western Reserve School of Medicine, Cleveland, OH, United States

**Keywords:** locally advanced rectal cancer, deficient mismatch repair, surgery, chemotherapy, chemoradiotherapy, progression-free survival, overall survival

## Abstract

**Background:**

For locally advanced rectal cancer (LARC) with a deficient mismatch repair/microsatellite instability-high (dMMR/MSI-H), particularly in patients not eligible for immunotherapy, the optimal treatment remains undetermined. This study was to evaluate the efficacy and safety of surgery, surgery and chemotherapy, surgery and chemoradiotherapy, in patients with LARC harboring dMMR/MSI-H.

**Methods:**

Patients included from three university centers between August 1, 2012 and March 1, 2023, were categorized into three treatment groups: surgery *vs*. surgery + chemotherapy *vs*. surgery + chemoradiotherapy. The primary endpoint was overall survival (OS), with secondary endpoints of progression-free survival (PFS), local recurrence (LR), distant metastasis (DM), and toxicity. The Kaplan-Meier method was utilized to analyze OS and PFS; competing risk methods were employed to evaluate rates of LR and DM. Adjustments were performed utilizing inverse probability of treatment weighting (IPTW) and overlap weighting (OW) based on propensity score, employing logistic regression model. The Cox proportional hazards model was applied for both univariate and multivariate analyses to assess prognostic factors influencing patient OS and PFS.

**Results:**

A total of 119 patients were included, with 45 patients (37.8%) receiving surgery alone, 32 (26.9%) receiving surgery + chemotherapy, and 42 (35.3%) undergoing surgery + chemoradiotherapy. In both the unadjusted cohort and after IPTW and OW adjustments, the surgery alone group (*vs*. surgery + chemoradiotherapy) had improved OS, PFS, LR, but no significant differences in DM. However, no statistical difference was found between the surgery *vs*. surgery + chemotherapy groups in OS, PFS, and DM, except for significant differences in LR. Similar results were found in both neoadjuvant and adjuvant treatment cohorts. No adverse events of grade 5 occurred.

**Conclusion:**

This study suggests surgery alone (without chemotherapy and/or radiotherapy) may be an optimal treatment for LARC patients with dMMR/MSI-H, particularly in those who cannot tolerate or access immunotherapy. The results of this study may be used to power a randomized trial for the approaches.

## 1 Introduction

Rectal cancer is one of most common malignancies and has an annual incidence of approximately 732,000 cases worldwide (1). Locally advanced rectal cancer (LARC), defined as clinical tumor stage 3–4 or clinical lymph node positive disease, accounts for a large proportion of rectal cancers. Approximately 10% of rectal cancers are deficient DNA mismatch repair (dMMR)/microsatellite instability-high (MSI-H) (2), more commonly seen in those < 50 years of age (3, 4).

Over a long period of time, the standard treatment strategy for patients with LARC harboring either dMMR/MSI-H or proficient DNA mismatch repair (pMMR)/microsatellite stability (MSS), as per the ESMO and NCCN guidelines, involves multimodal therapy based on total neoadjuvant therapy (TNT), with chemoradiotherapy and then resection (2, 5–8). There may be several disadvantages to trimodality therapy for these patients: (1) Patients who undergo trimodality therapy may have life-threatening perioperative complications such as anastomotic leakage, poor healing, as well as long-term impairment in urinary, anal, fertility, and sexual functional (9, 10). (2) The distant metastatic rate remains high, reaching about 20% at 3 years (11–13). (3) dMMR/MSI-H cancers may not respond well to chemotherapy or chemoradiotherapy, as they have low rates of complete response (14, 15). Thus, some clinicians argue that LARC with dMMR/MSI-H should be treated with surgery alone, rather than multimodal therapy.

Based on these considerations, and recognizing that surgery with or without chemo(radio)therapy remains an important treatment option for a subset of dMMR/MSI-H LARC patients who are ineligible for immunotherapy, even in the current immunotherapy era, we conducted a multicenter retrospective cohort study to evaluate outcomes with these three approaches. We hypothesize that outcomes are similar among the three approaches. The findings could serve as a valuable reference for further randomized controlled trials, which are essential to investigate and refine optimal treatment strategies beyond traditional chemotherapy or chemoradiotherapy in this context.

## 2 Patients and methods

### 2.1 Eligible patients

**Figure 1:**
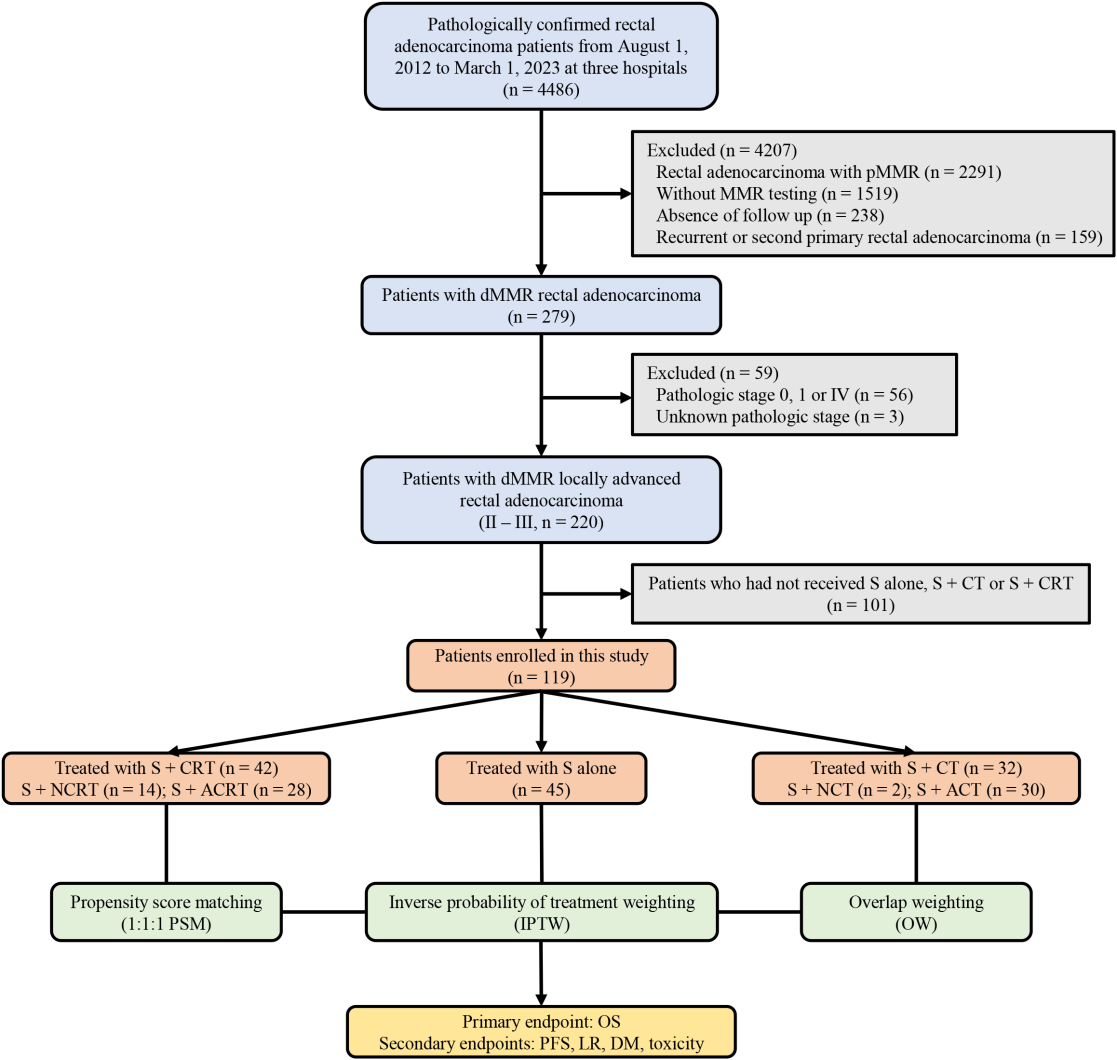
A detailed overview of the specific inclusion process for this study. pMMR, proficient DNA mismatch repair; dMMR, deficient mismatch repair; S+CT, surgery plus chemotherapy; S+CRT, surgery plus chemoradiotherapy; S+NCRT, surgery plus neoadjuvant chemoradiotherapy; S+ACRT, surgery plus adjuvant chemoradiotherapy; S, surgery; S+NCT, surgery plus neoadjuvant chemotherapy; S+ACT, surgery plus adjuvant chemotherapy; PSM, propensity score matching; IPTW, inverse probability of treatment weighting; OW, overlap weighting; OS, overall survival; PFS, progression-free survival; LR, local recurrence; DM, distant metastasis.

This retrospective cohort study included patients with LARC harboring dMMR/MSI-H and treated with surgery ± chemo(radio)therapy at Tianjin Medical University Cancer Institute and Hospital, Tianjin Union Medical Center of Nankai University and Tongji Hospital of Huazhong University of Science & Technology from August 1, 2012 to March 1, 2023. The inclusion criteria were as follows: (1) pathologically confirmed rectal adenocarcinoma; (2) dMMR/MSI-H determined using immunohistochemistry (IHC) or polymerase chain reaction (PCR) testing; (3) clinically diagnosed with LARC (T3-4N0 or T1-4N+) according to the AJCC Cancer Staging Eighth Edition; (4) underwent surgery and received either chemo(radio)therapy or not; (5) treated with long course/conventionally fractionated radiotherapy. Patients were excluded if they had recurrent or second primary rectal adenocarcinoma; lacked follow-up or had incomplete data collection; had discordant dMMR/MSI-H testing, or treated with short course radiotherapy. **Figure 1** provides a detailed overview of the specific inclusion process for this study. This study adhered to the ethical principles of the Helsinki Declaration and obtained approval from the independent ethics committee of three hospital centers.

### 2.2 MMR status determination and analyses

MMR status with IHC evaluated the expression of MMR proteins-MLH1, MSH2, MSH6, and PMS2 (16). The absence of any one of these proteins in the tumor classified the patient as dMMR. Additionally, microsatellite instability status was evaluated with PCR, involved analyzing the stability of two mononucleotide (BAT25 and BAT26) and three dinucleotide repeats loci (D5S346, D2S123, and D17S250) in both normal rectal mucosa and tumor tissue. Two or more loci out of the five were found to be unstable (MSI-H), the patient was classified as dMMR. If one locus was unstable (MSI-L) or all five loci were stable (MSS), the patient was categorized as proficient (pMMR) (17, 18).

### 2.3 Treatments

All patients were treated with definitive-intent surgery, which included Dixon, Miles, Hartmann, Total proctococectomy, or Intersphincteric resection. Long course/conventionally fractionated radiotherapy was administered with three-dimensional conformal radiotherapy or intensity-modulated radiotherapy, with a total dose ranging from 45.0 to 50.4 Gy delivered in 25 to 28 daily fractions. The clinical target volume (CTV) of radiotherapy encompassed primary rectal carcinoma and lymphatic drainage areas such as the mesorectum, internal iliac, and presacral lymph nodes. Chemotherapy cycles were administered every 21 days, and the median number of chemotherapy cycles was approximately 6 (interquartile range [IQR], 4-8). If patients received concurrent chemoradiotherapy, fluorouracil-based monotherapy was administered concurrently with radiotherapy. A subset analysis was performed with neoadjuvant treatment and adjuvant treatment. The interval between neoadjuvant therapy and surgery was about 6–8 weeks, and the interval between adjuvant therapy and surgery was approximately 4–6 weeks.

### 2.4 Endpoints

The primary endpoint of this study was overall survival (OS), defined as the time from treatment initiation to death or the last follow-up. Secondary endpoints included: (1) progression-free survival (PFS), defined as the duration from treatment initiation to disease progression (based on RECIST 1.1) or last follow up for censored patients; (2) local recurrence, defined as local disease progression (based on RECIST 1.1) after treatment initiation; (3) distant metastasis, defined as distant disease progression (based on RECIST v1.1) after treatment initiation; and (4) Common Terminology Criteria for Adverse Events (CTCAE v4.0) grade toxicity, based on multidisciplinary evaluation.

### 2.5 Statistical analysis

Categorical variables were compared using the χ2 test and Fisher’s exact test. OS and PFS were generated using the Kaplan-Meier method, and comparisons between groups were conducted via the log-rank test. The cumulative incidence of LR and DM were compared using doubly robust multivariable Fine and Gray regression models, enabling estimation of the subdistribution hazard function that models the hazard function in the presence of competing risks. All-cause mortality was considered a competing risk for LR and DM (19, 20). Univariate analyses (UVA) and multivariate analyses (MVA) were conducted using the Cox proportional hazards model to assess the prognostic factors influencing patient OS and PFS. Variables significant in the univariate analysis were considered for inclusion in the final multivariate model. Variables with a *p*-value < 0.2 were retained in the final multivariate model. Statistical analyses were conducted using R version 4.3.2 (R Foundation for Statistical Computing). In this study, the Benjamini-Hochberg correction was applied to adjust for multiple testing of pairwise comparisons of survival curves. Both these comparisons and other statistical analyses were considered statistically significant if the *p* value was < 0.05.

### 2.6 PSM, IPTW, and OW adjustments

To minimize data biases and confounding factors in terms of baseline characteristics, we utilized the multivariable logistic regression model approach described by Rubin and Rosenbaum to calculate propensity scores for Propensity score matching (PSM), inverse probability of treatment weighting (IPTW), and overlap weighting (OW) (21, 22). Variables involved in the regression model included gender (male *vs.* female), age (≤ 60 *vs.* > 60), pathological stage (II *vs.* III), distance from anal verge (≤ 5 cm *vs.* > 5 cm), differentiation (poorly *vs.* moderately *vs.* well), resection status (R0 *vs.* R1), and lymphovascular or neural invasion (negative *vs*. positive). Collinearity was tested using variance inflation factor to ensure the independence of each variable included in the regression model. The collinearities within the overall cohort and the adjuvant cohort were illustrated in **Supplementary Figure 1**. PSM included a logistic regression model and a 1:1:1 ratio matching with nearest-neighbor matching and a caliper of 0.2 times the standard deviation of the propensity score’s logit. For IPTW and OW, stabilized weights were calculated from the propensity score and used as weights (23). Standardized mean difference (SMD) was used to assess the balance of baseline covariates between treatment groups in the adjusted sample with that in the unadjusted sample. SMD values less than 0.2 indicated high levels of covariate balances. The SMD values within the overall cohort and the adjuvant cohort were illustrated in **Supplementary Figure 2**. Because PSM yielded inferior SMD results compared to IPTW and OW, IPTW and OW were ultimately selected as the preferred methods.

## 3 Results

### 3.1 Patient baseline characteristics

Between August 1, 2012 to March 31, 2023, 4486 patients were screened for MMR testing and eligibility, and 119 patients with dMMR/MSI-H were included. Among included patients, 45 patients (37.8%) underwent surgery alone, 32 patients (26.9%) received surgery + chemotherapy, included 2 and 30 patents who underwent neoadjuvant and adjuvant chemotherapy, as well as 42 patients (35.3%) received surgery + chemoradiotherapy, included 14 and 28 patents who underwent neoadjuvant and adjuvant surgery + chemoradiotherapy, respectively. The median follow-up periods for the three groups were 46.0 months (IQR, 42.5-88.6), 45.5 months (IQR, 28.3-93.0), and 47.9 months (IQR, 29.7-77.0) respectively.

In the unadjusted cohort, significant imbalances were observed in nearly half of patient baseline characteristics. In contrast, this cohort exhibited a good balance in patents baseline characteristics after both IPTW and OW adjustments (**Table 1**). Similar to the overall cohort, the postoperative adjuvant treatment cohort exhibited balanced patient baseline characteristics after IPTW and OW adjustments. Treatment characteristics of the overall cohort were outlined in **Table 2**. However, IPTW and OW adjustments could not be conducted for the neoadjuvant treatment cohort due to its small sample size. The MMR protein defect style of the overall cohort, adjuvant treatment cohort, and neoadjuvant treatment cohort were depicted in **Supplementary Figure 3**.

**Table 1:** Baseline demographic and clinical characteristics for patients received surgery alone, surgery plus CT, and surgery plus CRT.

Characteristics	Unadjusted cohort						IPTW-adjusted cohort		OW-adjusted cohort	
	All (N = 119), n (%)	Groups			SMD	*P*	SMD	*P*	SMD	*P*
		S alone (N = 45), n (%)	S + CT (N = 32), n (%)	S + CRT (N = 42), n (%)						
Gender					0.074	0.879	0.081	0.882	0.067	0.924
Male	64 (53.8)	25 (55.6)	16 (50.0)	23 (54.8)						
Female	55 (46.2)	20 (44.4)	16 (50.0)	19 (45.2)						
Age (years)					0.022	**0.022**	0.033	0.972	0.054	0.958
≤60	61 (51.3)	16 (35.6)	18 (56.3)	27 (64.3)						
>60	58 (48.7)	29 (64.4)	14 (43.8)	15 (35.7)						
Pathological stage^†^					0.820	**0.0001**	0.043	0.959	0.080	0.896
II	69 (58.0)	37 (82.2)	20 (62.5)	12 (28.6)						
III	50 (42.0)	8 (17.8)	12 (37.5)	30 (71.4)						
Distance from anal verge (cm)					0.358	0.078	0.124	0.710	0.038	0.982
≤5	37 (31.1)	13 (28.9)	6 (18.8)	18 (42.9)						
>5	82 (68.9)	32 (71.1)	26 (81.2)	24 (57.1)						
Differentiation					0.368	0.181	0.129	0.976	0.070	0.996
Poorly	21 (17.6)	5 (11.1)	10 (31.2)	6 (14.3)						
Moderately	96 (80.7)	39 (86.7)	22 (68.8)	35 (83.3)						
Well	2 (1.7)	1 (2.2)	0	1 (2.4)						
Resection status					0.523	**0.003**	0.292	0.221	0.0001	1.000
R0	109 (91.6)	45 (100.0)	25 (78.1)	39 (92.9)						
R1	10 (8.4)	0	7 (21.9)	3 (7.1)						
Lymphovascular or neural invasion					0.324	0.098	0.098	0.795	0.048	0.970
Negative	110 (92.4)	42 (93.3)	27 (78.1)	41 (97.6)						
Positive	9 (7.6)	3 (6.7)	5 (15.6)	1 (2.4)						

After weighting, a single individual no longer represents a single data entity and thus raw counts are not reported after weighting. Bold face denotes *P* value < 0.05.

IPTW, Inverse probability of treatment weighting; O, Overlap weighting; S, Surgery; CT, Chemotherapy; S + CT, Surgery plus chemotherapy; S + CRT, Surgery plus chemoradiotherapy; SMD, Standardized mean Difference; cm, Centimeters.

^†^American Joint Committee on Cancer (AJCC) 8th edition.

**Table 2:** Treatment characteristics for patients received surgery alone, surgery plus CT, and surgery plus CRT.

Characteristics	Unadjusted cohort					
	All (N = 119), n (%)	Groups			F/χ2	*P*
		S alone (N = 45), n (%)	S + CT (N = 32), n (%)	S + CRT (N = 42), n (%)		
Surgical style					12.765	**0.047**
Dixon	81 (68.1)	34 (75.6)	25 (78.1)	22 (52.4)		
Miles	25 (21.0)	4 (8.9)	6 (18.8)	15 (35.7)		
Hartmann	11 (9.2)	6 (13.3)	1 (3.1)	4 (9.5)		
Others^†^	2 (1.7)	1 (2.2)	0	1 (2.4)		
CT					107.420	**0.0001**
Yes	71 (59.7)	0	32 (100.0)	39 (92.9)		
No	48 (40.3)	45 (100.0)	0	3 (7.1)		
Drugs agent					114.68	**0.0001**
Single agent	5 (4.2)	0	3 (9.4)	2 (4.8)		
Double agent	63 (52.9)	0	26 (81.3)	37 (88.1)		
None	48 (40.3)	45 (100.0)	0	3 (7.1)		
Unknown	3 (2.5)	0	3 (9.4)	0		
CT regimens					114.77	**0.0001**
Fluoropyrimidine based	6 (5.0)	0	3 (9.4)	3 (7.1)		
Oxaliplatin based	16 (13.4)	0	8 (25.0)	8 (19.0)		
Fluoropyrimidine + oxaliplatin based	46 (38.7)	0	18 (56.3)	28 (66.7)		
None	48 (40.3)	45 (100.0)	0	3 (7.1)		
Unknown	3 (2.5)	0	3 (9.4)	0		

Bold face denotes *P* value < 0.05.

S, Surgery; CT, Chemotherapy; S + CT, Surgery plus chemotherapy; S + CRT, Surgery plus chemoradiotherapy; Fluoropyrimidine based CT, Fluoropyrimidine or capecitabine single agent chemotherapy; Oxaliplatin based CT, XELOX chemotherapy; Fluoropyrimidine + oxaliplatin based CT, FOLFOX, FOLFIRI or sequential FOLFOX, FOLFIRI, to XELOX chemotherapy.

^†^Other surgical styles were total proctococectomy (n = 1) and intersphincteric resection (n = 1).

### 3.2 Outcomes overall cohort

**Figure 2:**
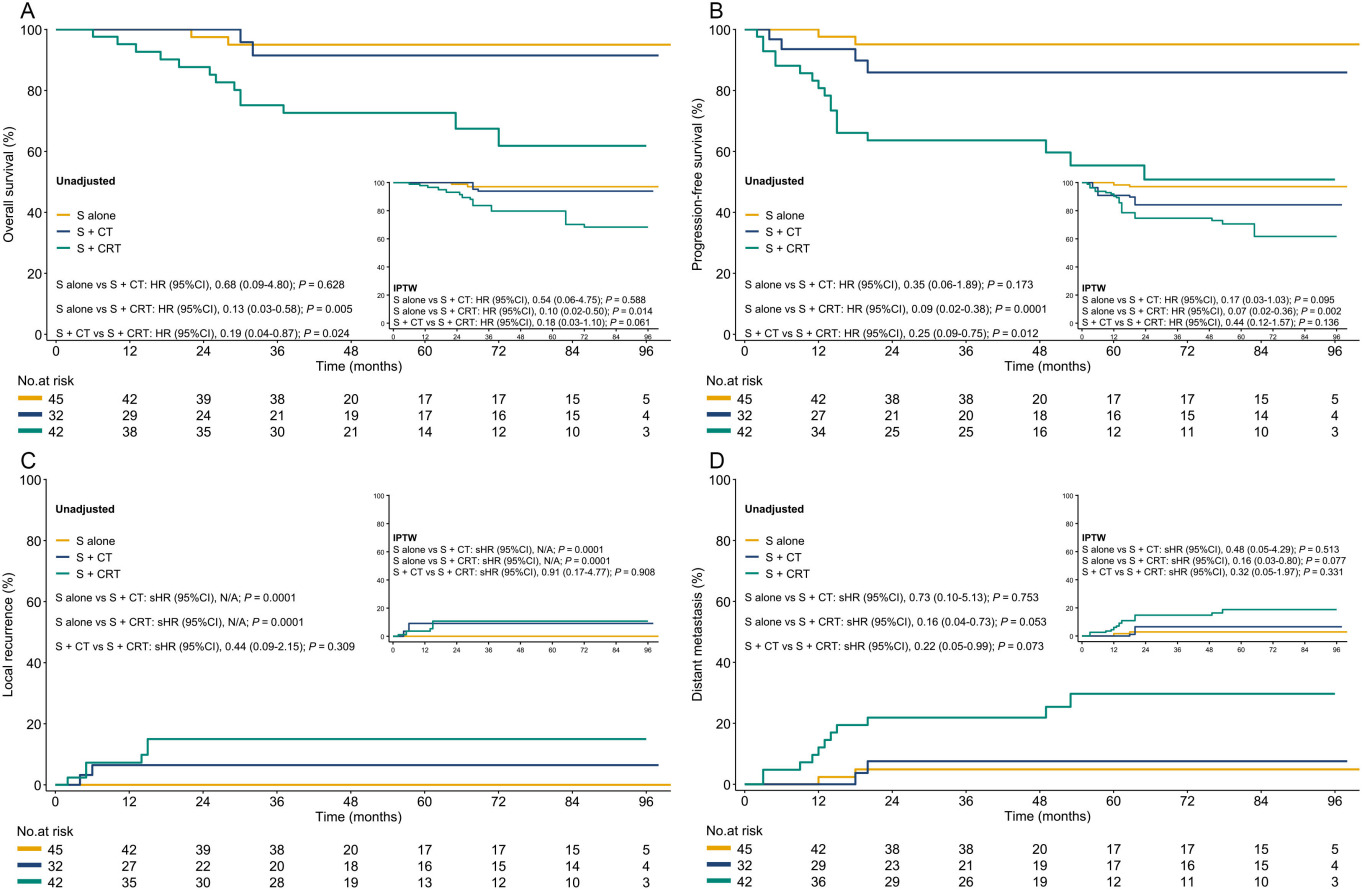
The OS, PFS, LR, and DM in both the unadjusted and IPTW-adjusted cohorts. **(A)**: OS; **(B)** PFS; **(C)** LR; **(D)** DM. S, surgery; S+CT, surgery plus chemotherapy; S+CRT, surgery plus chemoradiotherapy; HR, hazard ratio; IPTW, inverse probability of treatment weighting; sHR, subdistribution hazard ratio.

In both the unadjusted cohort and after IPTW and OW adjustments, patients in the surgery alone (*vs*. surgery + chemoradiotherapy) was associated with improved in OS, PFS, and LR, but no significant differences in DM. However, no statistically significant difference was found between the surgery alone *vs*. surgery + chemotherapy groups in OS, PFS, and DM, except for significant differences in LR (**Figure 2**, **Supplementary Figure 4**). We conducted a subgroup analysis based on treatment modalities, where surgery alone was associated with improved outcomes *vs*. the surgery + adjuvant chemoradiotherapy group in the unadjusted cohort. However, no statistical difference was found among these three groups in both IPTW (**Supplementary Figure 5**) and OW adjustments. Although only 13.4% (16/119) included patients received neoadjuvant therapy, similar results were seen in the surgery *vs*. neoadjuvant chemoradiotherapy group comparison (**Supplementary Figure 6**). After adjustments, there was no statistically significant difference in outcomes in the surgery *vs*. the surgery + chemotherapy groups, except for significant differences in LR.

### 3.3 Prognostic factors of OS and PFS

**Figure 3:**
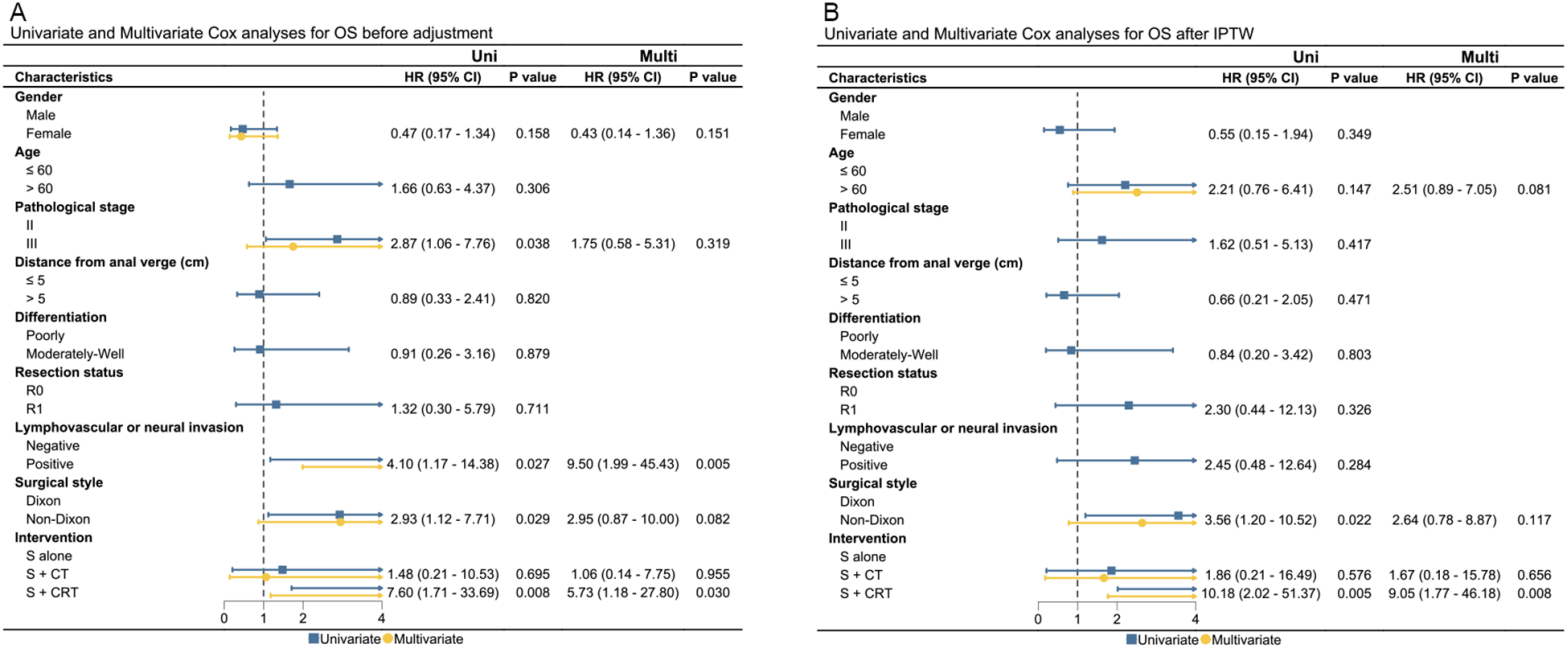
The UVA and MVA analysis for clinical factors affecting OS in both the unadjusted and IPTW-adjusted cohorts. **(A)** OS before adjustment; **(B)** OS after IPTW. OS, overall survival; Uni, univariate analysis; Multi, multivariate analysis; HR, hazard ratio; CI, confidence interval; IPTW, inverse probability of treatment weighting; HR, hazard ratio; cm, centimeter.

**Figure 4:**
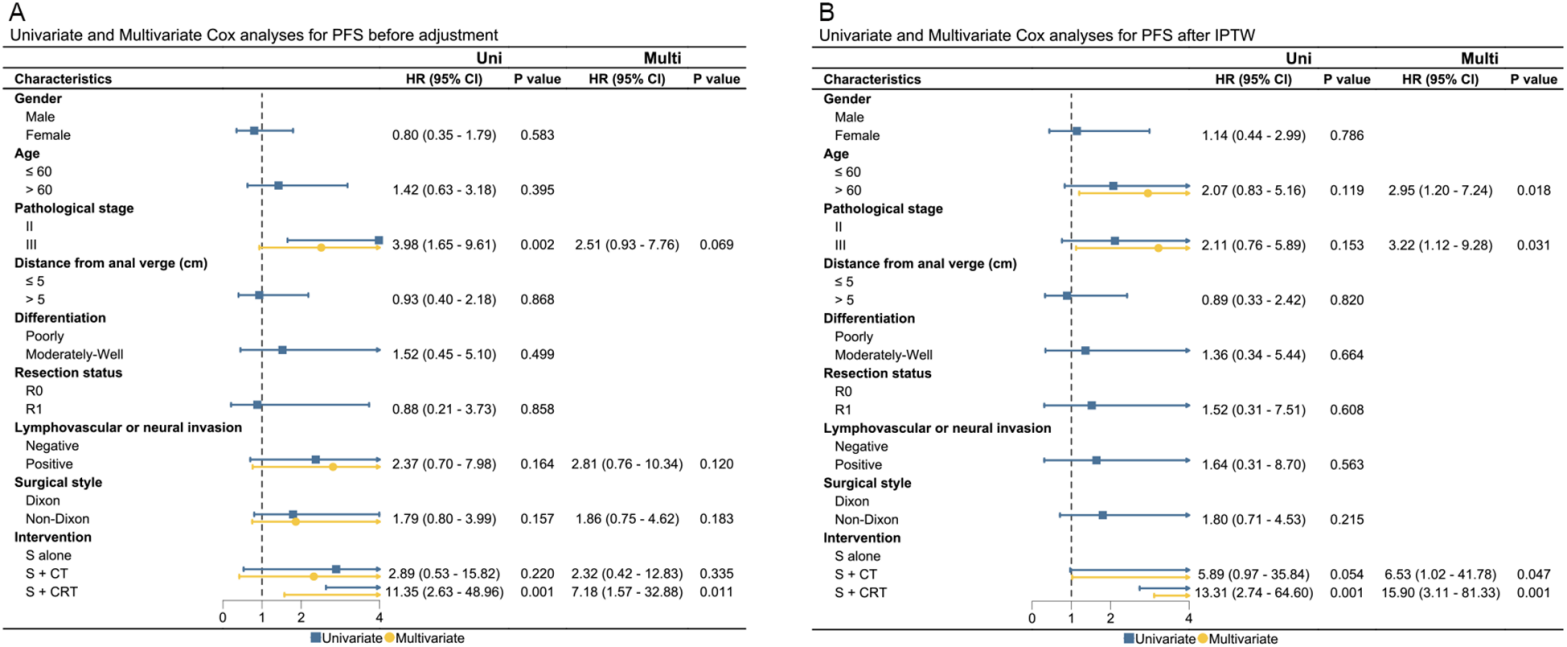
The UVA and MVA analysis for clinical factors affecting PFS in both the unadjusted and IPTW-adjusted cohorts. **(A)** PFS before adjustment; **(B)** PFS after IPTW. PFS, progression-free survival; Uni, univariate analysis; Multi, multivariate analysis; HR, hazard ratio; CI, confidence interval; IPTW, inverse probability of treatment weighting; HR, hazard ratio; cm, centimeter.

Presence of pathological stage III, lymphovascular or neural invasion, non-Dixon surgery style, and surgery + chemo(radio)therapy were significantly associated with worse OS or PFS in the unadjusted cohort. After adjustments using IPTW and OW, only surgery + chemoradiotherapy were found to be correlated with worse OS and PFS after IPTW and OW adjustments. Results of UVA and MVA for clinical factors affecting OS and PFS are presented in **Figures 3**, **4**, and **Supplementary Figure 7**. Pathological stage III and surgery + chemoradiotherapy were an adverse prognostic factor for OS and PFS in both UVA and MVA in both unadjusted and adjusted matching for adjuvant treatment cohort (**Supplementary Figure 8**), while only surgery + chemoradiotherapy was adverse prognostic factor for OS and PFS in both UVA and MVA in the unadjusted cohort for neoadjuvant treatment cohorts.

### 3.4 Toxicities

The most common treatment-emergent adverse events of any grade ≥ 5% were nausea and vomiting (51/119, 42.9%), diarrhea (48/119, 40.3%), hepatotoxicity (33/119, 27.7%), neutropenia (27/119, 22.7%), neurotoxicity (22/119, 18.5%), and leukopenia (17/119, 14.3%). The grade 3–4 treatment-emergent adverse events were diarrhea (18/119, 15.1%), leukopenia (10/119, 8.4%), neurotoxicity (9/119, 7.6%), nausea and vomiting (4/119, 3.4%), and thrombocytopenia (4/119, 3.4%). All surgery-related adverse events were grade 1–2, including colostomy and subsequent reversal (4/119, 3.3%), anastomotic leakage (3/119, 2.5%), postoperative bleeding (5/119, 4.2%), and urogenital dysfunction (6/119, 5.0%). No adverse events of grade 5 occurred. There was no significant difference among the three groups with respect to any grade and grade 3–4 adverse events.

## 4 Discussion

To the best of our knowledge, few retrospective studies have evaluated the suitability of chemotherapy or chemoradiotherapy in patients with LARC harboring dMMR/MSI-H, but the contradictory results have arisen from a comparison of LARC patients between those with dMMR/MSI-H and pMMR/MSS (24–27). This difference may partly be attributable to the low incidence of dMMR/MSI-H in rectal cancer and thereby to the small sample size (28). Importantly, our results suggested that there was no improvement in outcomes from the addition of chemotherapy or chemoradiotherapy. A possible biological explanation for these results may lies in the MMR protein biological function. In the absence of a functional MMR system, repair may only occur through the “base excision repair” system, a process that is less affected by the disequilibrium or methylation induced by chemotherapy or chemoradiotherapy (29–31). Therefore, identifying the optimal treatment strategy to improve the response of LARC patients with dMMR/MSI-H, particularly those who are not eligible for immunotherapy, is of utmost importance, necessitating further large-scale studies to refine the optimal treatment strategy.

Theoretically, neoadjuvant therapy results in downstaging, lymph node clearance, and clearance of lymphovascular involvement; however, only 13.4% (16/119) included patients received neoadjuvant therapy, while the majority underwent surgery combined with adjuvant chemoradiotherapy instead of the current neoadjuvant treatment era. We believe this may be due to two main factors. First, the treatment paradigm for LARC has evolved gradually over time—from surgery alone, to the incorporation of adjuvant therapy, and eventually to the widespread adoption of neoadjuvant strategies. The timing of this transition has varied across countries and regions (32–35). Although the utilization of neoadjuvant chemoradiotherapy has been gradually increasing in certain Chinese medical centers, the overall adoption of this treatment strategy occurred later in China than in some Western countries and may still lag behind levels seen in developed countries (33, 34). Second, dMMR/MSI-H tumors exhibit distinct biological behavior, including reduced sensitivity to chemo(radio)therapy, as reported in prior studies (14, 25, 36). As this resistance has become increasingly recognized, clinicians often prioritize surgery and reserve adjuvant therapy for patients with high-risk postoperative features. Importantly, we conducted a subgroup analysis based on treatment modalities, where surgery alone was associated with improved outcomes compared to surgery combined with (neo)adjuvant therapies, including chemotherapy or chemoradiotherapy in the unadjusted cohort. However, no statistically significant differences were observed among these three groups in both IPTW and OW adjustments.

Early studies on immunotherapy in rectal cancer patients without specific molecular characteristics showed disappointing results (37). Nevertheless, there is a growing interest in the use of immunotherapy and immune-based strategies for treating LARC with dMMR/MSI-H. We comprehensively reviewed published research on immunotherapy and immune-based strategies for LARC patients with dMMR/MSI-H by searching PubMed and the clinical trial database. The final search date was June 1, 2024, and resulted in the inclusion of a total of 24 articles (as detailed in **Supplementary Table 1**) (38–65).

Emerging evidence indicates the extraordinary response of immunotherapy in treating LARC with dMMR/MSI-H. Based on these data, the latest NCCN guideline (https://www.nccn.org/guidelines) provides the preferred treatment strategy for patients with LARC harboring dMMR/MSI-H, recommending the initiation of immunotherapy for patients who have not received prior immune checkpoint inhibitors (ICIs). It suggests adopting a W&W if a clinical complete response (cCR) is achieved. Otherwise, continue with chemoradiotherapy combined with or without surgery. However, neoadjuvant monotherapy with ICIs has shown cCR or pathological complete response (pCR) rates ranging from 37.5% to 100%, comparable to those achieved with neoadjuvant immune-based combination therapy, where cCR or pCR rates range from 60% to 100%. These results suggest that surgery may be essential for some LARC patients with dMMR/MSI-H who do not achieve cCR.

Currently, many questions remain unanswered, such as the optimal timing for initiating ICIs, the appropriate dosage of immunotherapeutic agents, the ideal duration of ICIs, and the optimal combination strategy of ICIs and chemotherapy or chemoradiotherapy before surgery. Additionally, distinguishing masses containing inflammatory cells, necrotic tissue, and/or fibrous tissues from those containing tumor cells poses a challenge. Furthermore, the OS and PFS outcomes for neoadjuvant monotherapy with ICIs have not yet been finalized. Nevertheless, these promising results, albeit primarily derived from small clinical series, provide optimism for the future. Therefore, the optimal treatment strategy of LARC with a dMMR/MSI-H has yet to be clearly defined, necessitating additional studies and efforts to refine the optimal treatment strategy.

For patients with LARC harboring dMMR/MSI-H who achieve cCR after ICIs monotherapy or combination therapy, the option of omitting surgery and proceeding with observation alone may offer the possibility of cure without functional impairment. However, resistance to ICIs is frequently observed in the neoadjuvant setting, with the rate ranging between 10% and 40% (37, 48, 50, 51, 64–69). This results in the forfeiture of the optimal surgical opportunity for these patients. Apart from the biological mechanisms underlying resistance to immunotherapy in LARC with dMMR/MSI-H, consistent with our study (8.4%), nearly 10% of cases can largely be attributed to misdiagnosis of MMR/MSI status (70). Therefore, the combined use of both IHC and PCR is recommended to prevent misdiagnosis of dMMR/MSI-H status. Importantly, there is no well-established biomarker for ICIs resistance in these patients. Next-generation molecular profiling may provide further insight into biologic underpinnings, including potential drivers of oncogenesis or antioncogesis (*e.g.*, BRAF V600E, AKT1, CDH1, PTEN, or PIK3CA) (71–74).

This study has several limitations that need to be acknowledged. To the best of our knowledge, this study comprises a large sample size of LARC harboring dMMR/MSI-H who have undergone surgery with or without chemo(radio)therapy as curative intent treatment and could provide useful indications for future prospective trials. Firstly, we acknowledge your observation that the sample size exceeds the recommended ten events per variable (EPV) guideline in logistic regression analysis. However, previous studies published in prestigious journal, have suggested that even lower ratios, such as five events per variable, have been employed successfully in small patient cohorts with rare diseases (73–77). Indeed, the appropriateness of EPV ratios is context-dependent and influenced by factors such as outcome variability, type of variables, and survival analysis considerations. Additionally, this study possesses the following characteristics: (1) it is an exploration study; (2) independent variables were screened prior to conducting multivariate regression; (3) the HR and 95% CI of the results exhibit normalcy; (4) goodness-of-fit statistics indicate successful modeling; (5) despite some instability, the results consistently reflect the characteristic under consideration. In recent years, numerous high-quality scholarly work have adopted similar analytical method (73–78).

Secondly, the retrospective nature of this study introduces inherent selection bias given its span over one decade. Although we attempted to adjust for confounding factors using PSM, IPTW, and OW, there may still be some uncontrolled potential biases and confounding factors, such as the majority of patients undergoing surgery plus adjuvant chemoradiotherapy instead of the current neoadjuvant treatment era. Thirdly, no patients in the present cohort receive ICIs. Fourth, MMR status in this study was mainly determined through IHC, with only a small number of patients undergoing dual testing with IHC and PCR, potentially leading to false-positive results (57). Among patients who underwent both IHC and PCR testing, we observed concordance rates consistent with previously reported levels ranging from 91.4% to 99.6% (79–82). Given this high level of agreement between the two methods, the likelihood of misclassification within our cohort is expected to be low. Fifth, we collected follow-up data using electronic medical records and telephone follow-ups, resulting in missing data related to treatment-related adverse events, thus precluding analysis of this data. In addition, none of included patients underwent detection of germline genes and confirmed a diagnosis of Lynch syndrome. Given the familial heritability of Lynch syndrome and the potential for multiple primary malignancies, we will continue to encourage subjects who are young or suspected of Lynch syndrome to undergo germline genetic testing for better long-term management and follow-up.

## 5 Conclusion

Neoadjuvant chemoradiotherapy followed by rectal surgery resection is a standard treatment for LARC, but it is unclear if this approach is ideal for dMMR/MSI-H patients. This multicenter retrospective cohort study suggests no improvement in outcomes from the addition of chemotherapy or chemoradiotherapy *vs*. surgery alone, and a possible detriment in outcomes with either neoadjuvant or adjuvant chemoradiotherapy. Although caution should be used in the interpretation of retrospective comparative effectiveness research, the results of this study may be used to support the use of surgery alone for select patients (particularly in those who cannot tolerate or access immunotherapy) and to power a randomized controlled trial to evaluate the different treatment approaches.

## Data Availability

The raw data supporting the conclusions of this article will be made available by the corresponding author (Meng M-B), without undue reservation.
