# Supplementary material for: The efficacy and safety of neoadjuvant and adjuvant chemo(radio)therapy combined with surgery in patients with locally advanced rectal cancer harboring defective mismatch repair system: a large-scale multicenter propensity score analysis
Source: Front Immunol. 2025 Jul 7;16:1626438. doi: 10.3389/fimmu.2025.1626438 (PMC12277327; doi:10.3389/fimmu.2025.1626438)
Supplement: Supplementary file 1 [file DataSheet1.pdf]

Supplementary Figure 1. The collinearities within the overall cohort and the adjuvant cohort. (A) the overall cohort; (B) the adjuvant cohort.

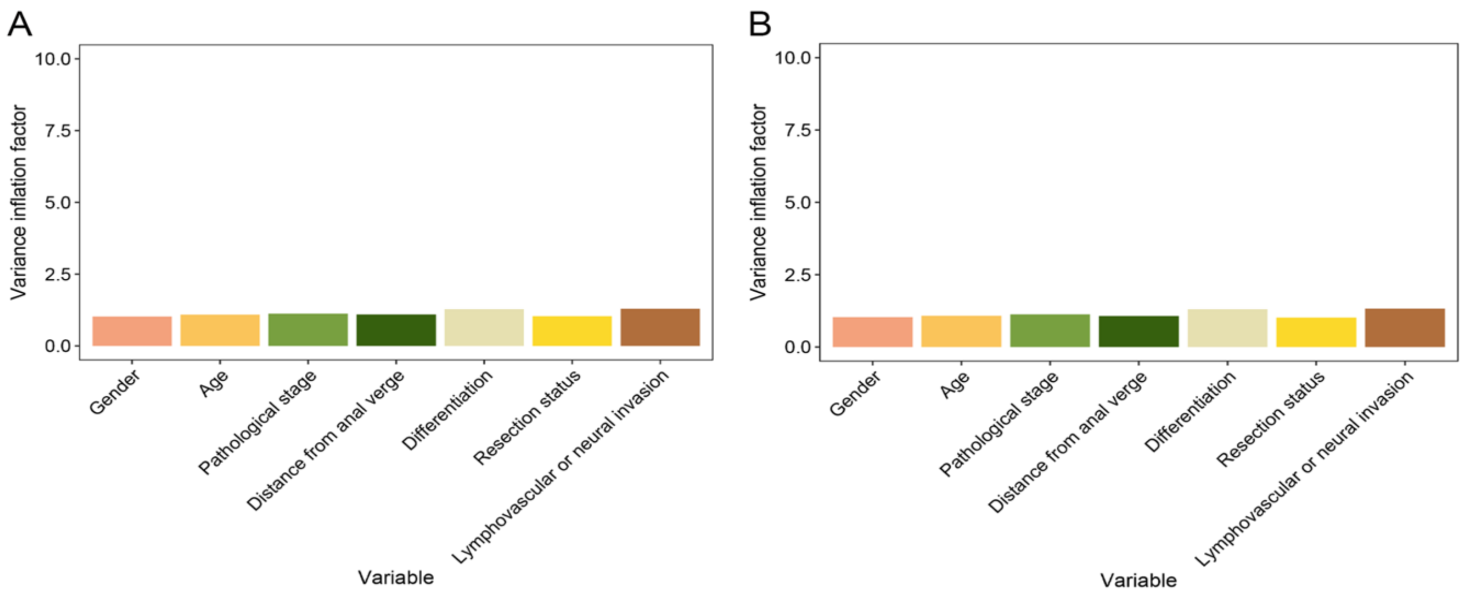

Supplementary Figure 2. The SMD values within the overall cohort and the adjuvant cohort. (A) the overall cohort; (B) the adjuvant cohort.

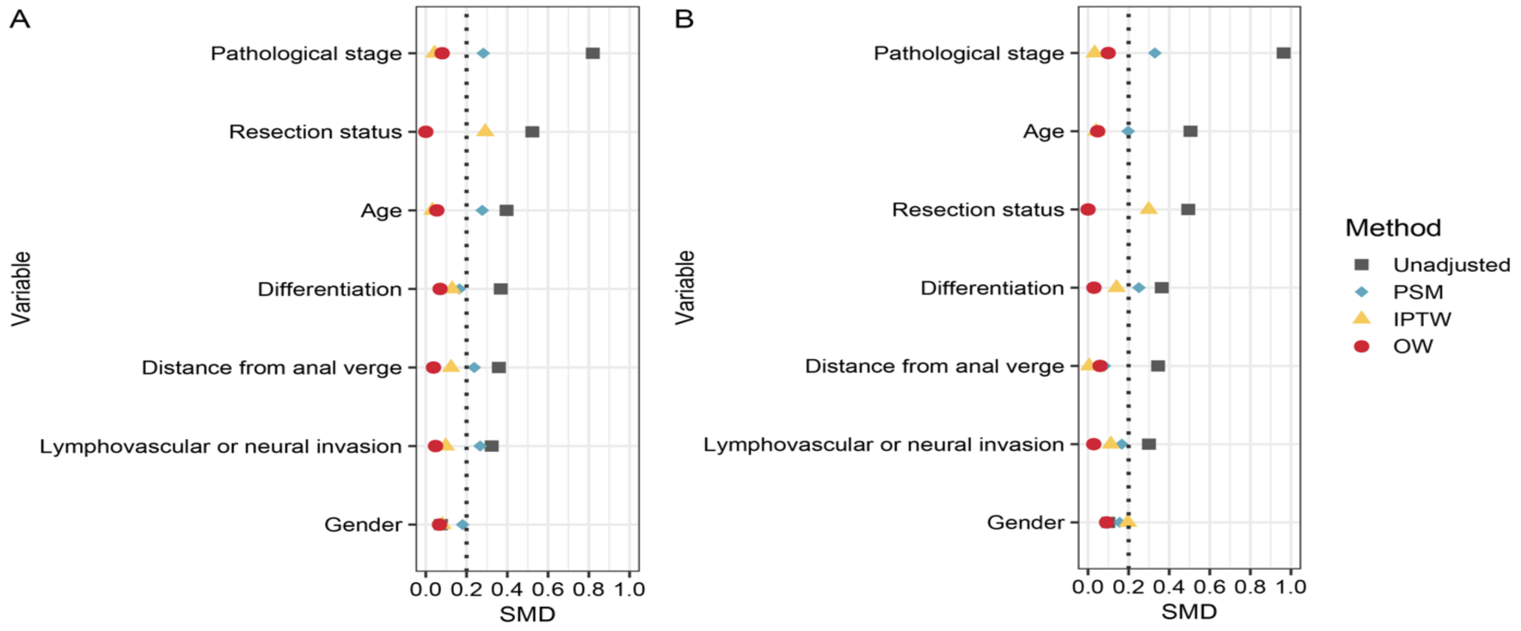

Supplementary Figure 3. The MMR protein defect style of the overall cohort, adjuvant treatment cohort, and neoadjuvant treatment cohort. (A) the overall cohort; (B) the adjuvant cohort; (C) the neoadjuvant cohort.

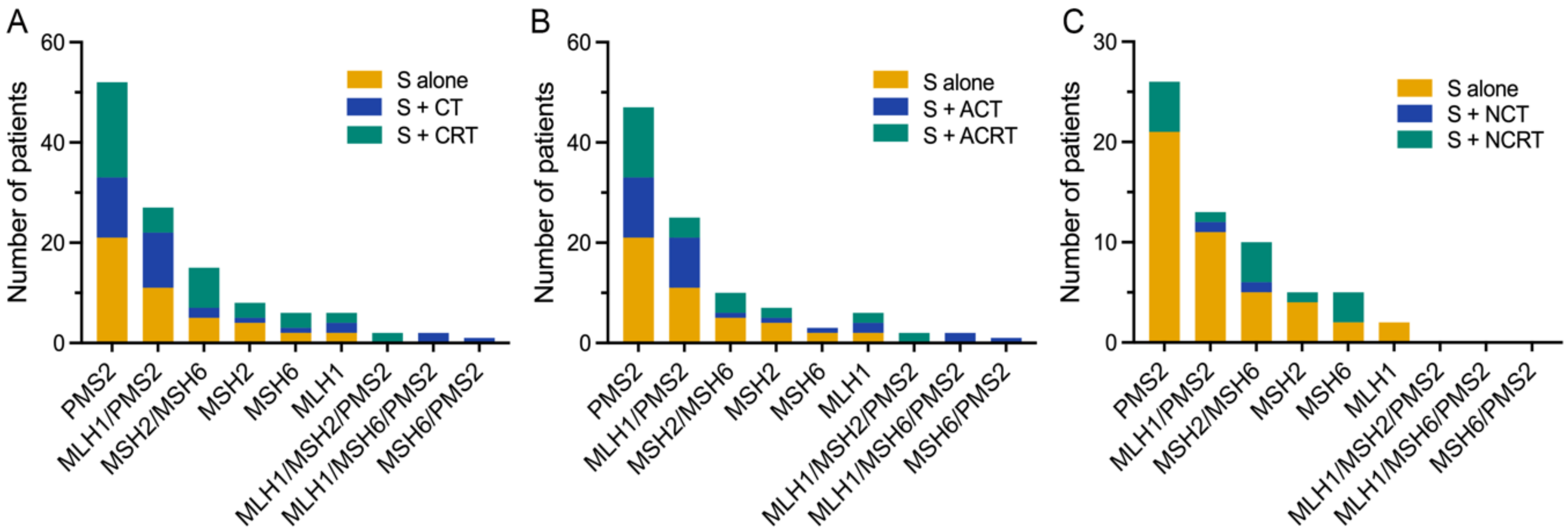

Supplementary Figure 4. The OS, PFS, LR, and DM in both the unadjusted and OW-adjusted cohorts. S: surgery; S+CT: surgery plus chemotherapy; S+CRT: surgery plus chemoradiotherapy; HR: hazard ratio; OW: overlap weighting; sHR: subdistribution hazard ratio. (A): OS; (B) PFS; (C) LR; (D) DM.

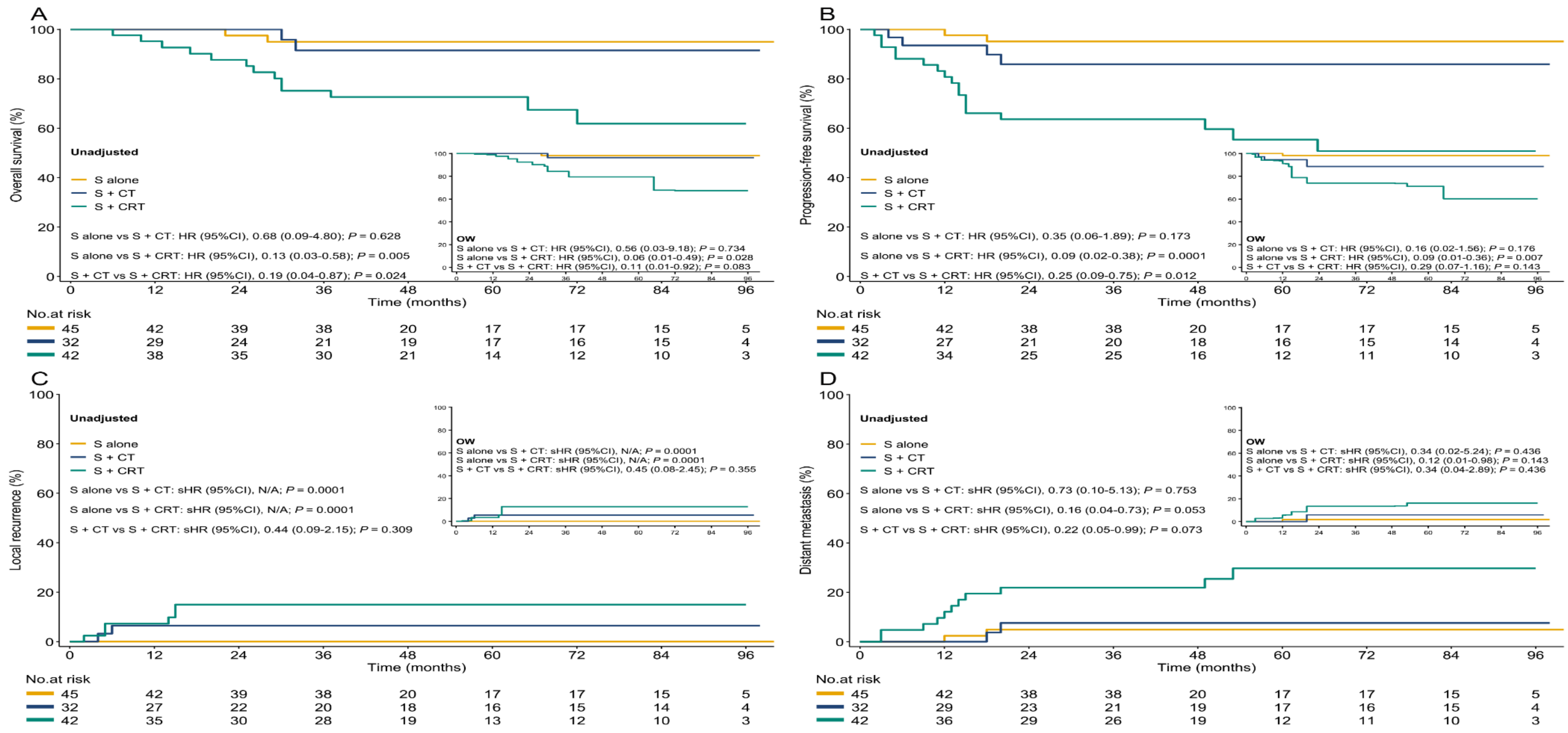

Supplementary Figure 5. The OS, PFS, LR, and DM in both the unadjusted and IPTW-adjusted cohorts for patients received surgery alone, surgery plus ACT, and surgery plus ACRT. (A): OS; (B) PFS; (C) LR; (D) DM.

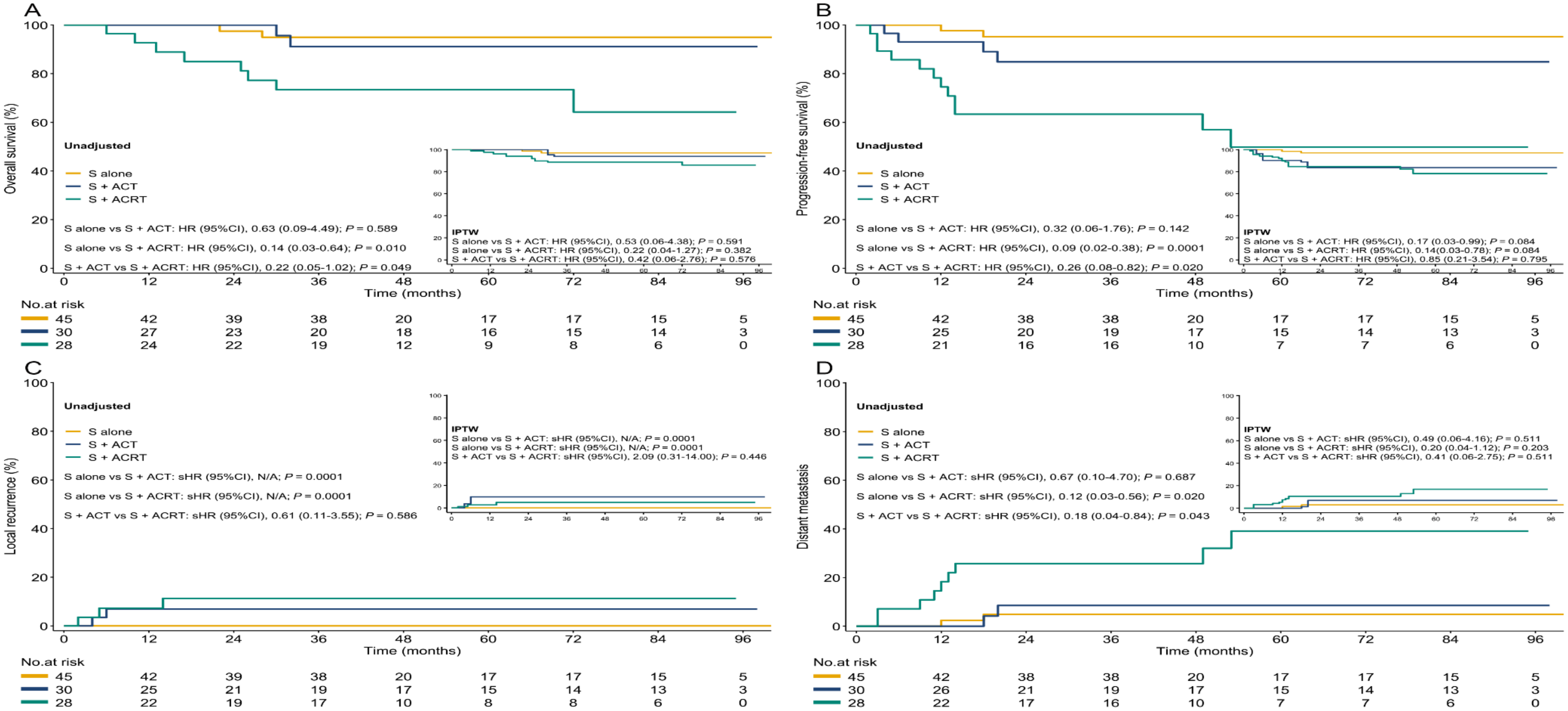

Supplementary Figure 6. The OS, PFS, LR, and DM for patients received surgery alone, surgery plus ACT, and surgery plus ACRT. (A): OS; (B) PFS; (C) LR; (D) DM.

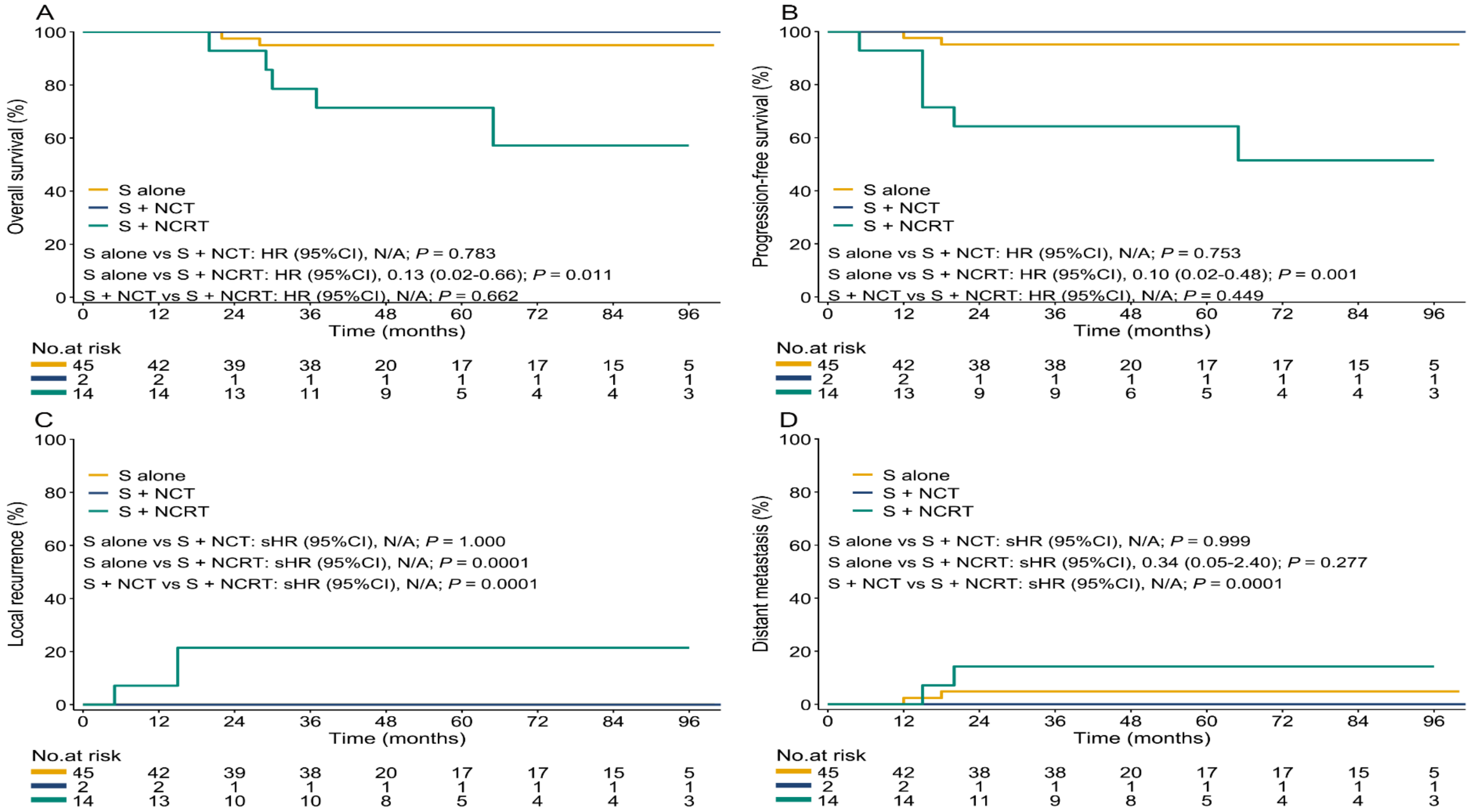

Supplementary Figure 7. The UVA and MVA analysis for clinical factors affecting OS in both the unadjusted and OW-adjusted cohorts. (A) OS; (B) PFS.

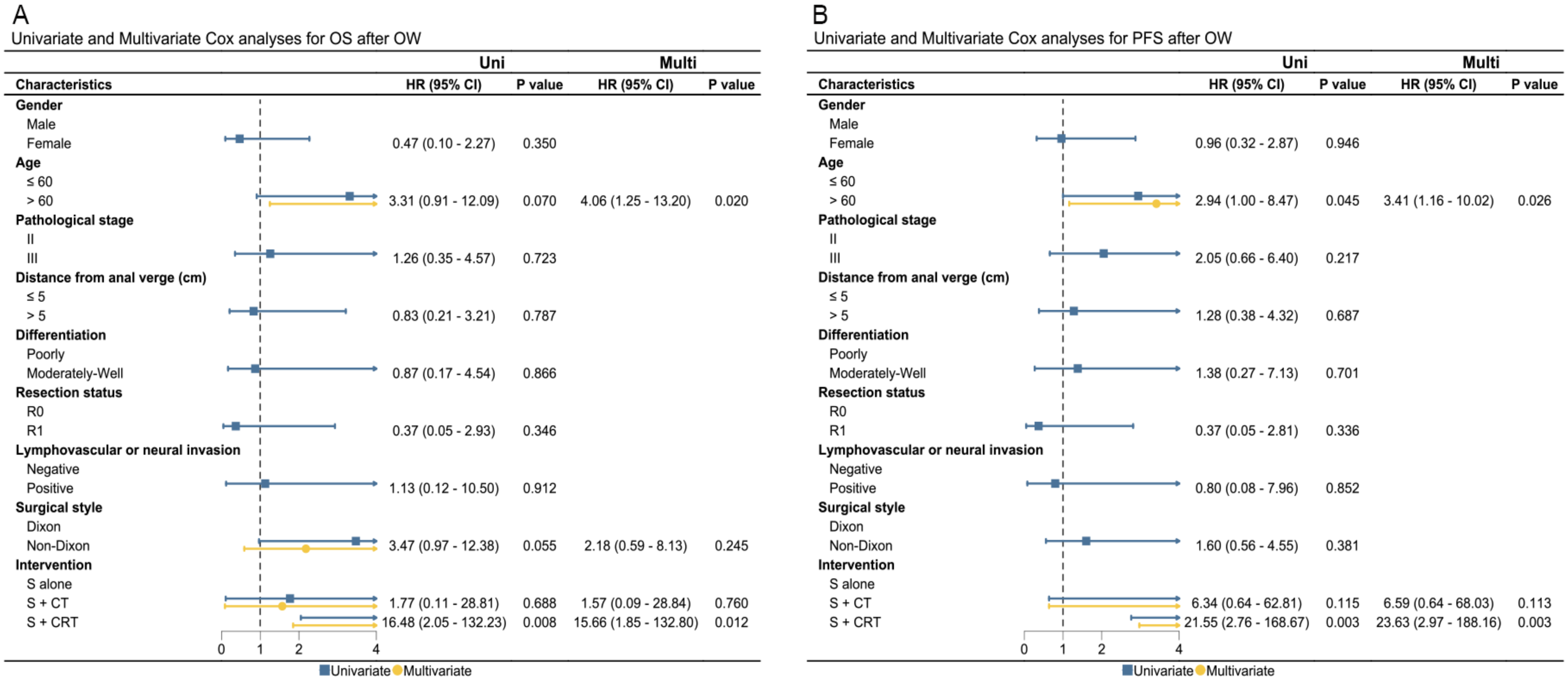

Supplementary Figure 8. The UVA and MVA analysis for clinical factors affecting OS and PFS in both the unadjusted and IPTW-adjusted for patients received surgery alone, surgery plus ACT, and surgery plus ACRT. (A-B) OS; (C-D) PFS.

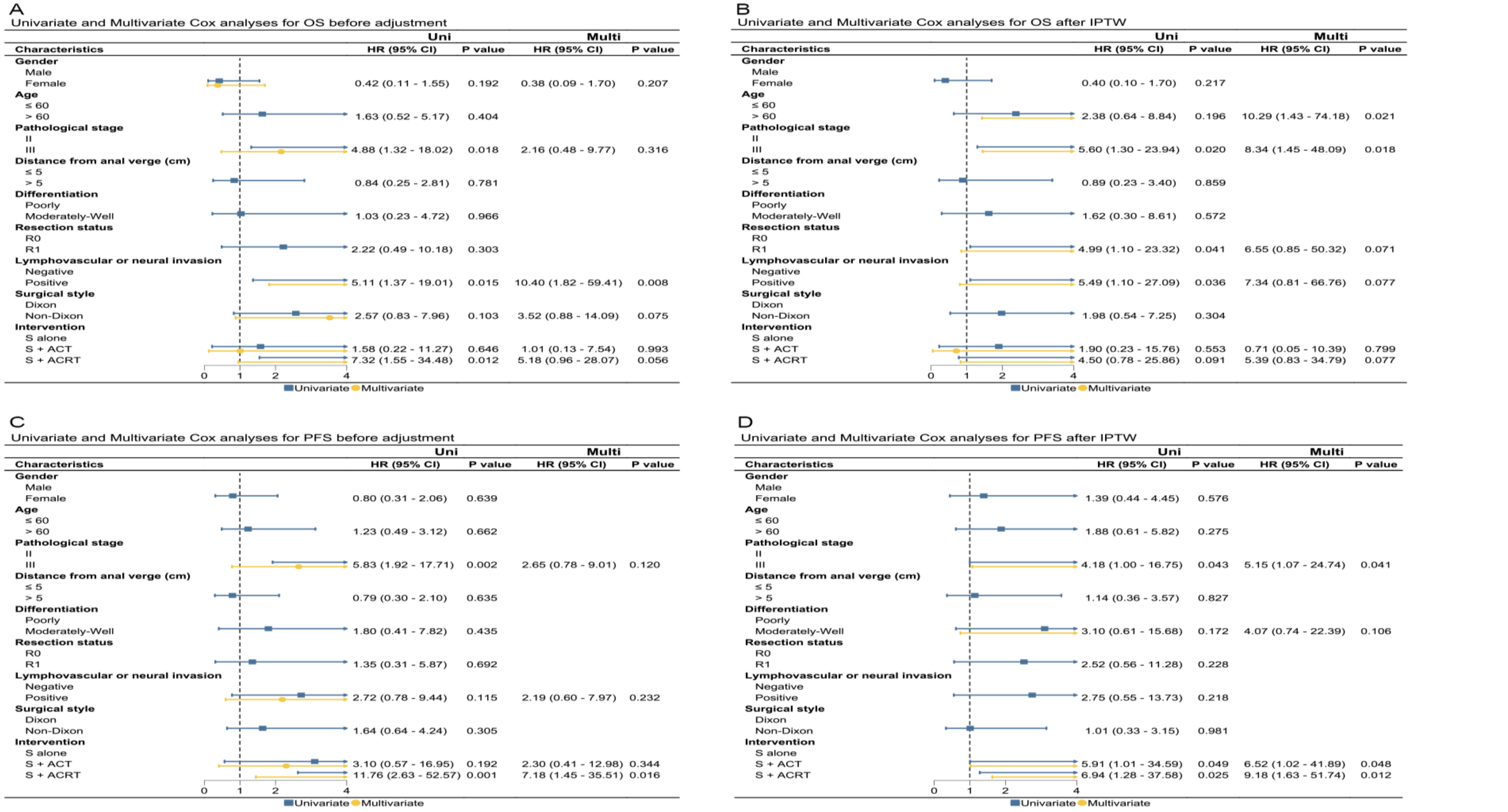

Supplementary Table 1. Response rates to immunotherapy-containing treatment regimens in LARC patients with dMMR/MSI-H.

Study (year) and references	Country	Study design	Sample size	Tumor stage	MMR detection	Treatment regimens	Follow-up (median, mths)	Tumor response	irAEs (CTCAE Grade ≥ 3)
Zhang, *et al.* (2019)[38]	China	Case series	2	Patient 1: $cT_4N_2M_0$; Patient 2: $rT_4N_2M_0$	IHC	Patient 1: Nivo × 6 cycles → TME; Patient 2: mFOLFOXIRI × 4 cycles → Nivo × 6 cycles → W&W	12	Patient 1: pCR rate: 100% (1/1); Patient 2: cCR rate: 100% (1/1)	None
Deng, *et al.* (2019)[39]	China	Pro, phase II	1	$cT_{3\text{-}4}N_0$ or $cT_xN_+M_0$	N/A	mFOLFOXIRI → Nivo → TME ± adjuvant CT	N/A	pCR rate: 100% (1/1)	N/A
Demisse, *et al.* (2020)[40]	USA	Case series	3	Patient 1: $cT_3N_1M_0$; Patient 2: $cT_3N_1M_0$; Patient 3: $cT_3N_2M_0$	IHC/PCR/NGS	Patient 1: Pembro × 11 cycles; Patient 2: FOLFOX → CRT → SD → (Ipi + Nivo) × 3 cycles → Nivo × 7 cycles; Patient 3: (FOLFOX + Pembro) × 7 cycles → TME	Patient 1: 17; Patient 2: 12; Patient 3: 10	pCR rate: 100% (3/3)	None
Liu, *et al.* (2020)[41]	China	Retro	1	$cT_3N_1M_0$	IHC + PCR/NGS	Nivo × 12 cycles → TME	N/A	pCR rate: 100% (1/1); TRG G_0 rate: 100% (1/1)	N/A
Zhang, *et al.* (2020)[42]	China	Case series	1	$cT_{3\text{-}4}N_0$ or $cT_xN_+M_0$	IHC + NGS	XELOX → (Sintili + Bev) × 4 cycles → Sintili × 1 cycle → TME	N/A	pCR rate: 100% (1/1)	None
Mans, *et al.* (2020)[43]	Belgium	Case series	1	$cT_3N_2M_0$	IHC/PCR/NGS	(Ipi + Nivo) × 3 cycles → TME → Nivo × 4 cycles	8.1	pCR rate: 100% (1/1)	N/A
Wang, *et al.* (2021)[44]	China	Retro	9	$cT_{3\text{-}4}N_0$ or $cT_xN_+M_0$	N/A	Anti-PD-1 Ab × 5 (3-7) cycles → W&W	15.6	cCR rate: 100% (9/9); 1-yr LRFS/DMFS/DFS rate: 100% (9/9)	N/A
Lin, *et al.* (2021)[45]	China	Pro, phase II	1	$cT_{3\text{-}4}N_0$ or $cT_xN_+M_0$	IHC	SCRT → (CAPOX + Camrelizu) × 2 cycles → TME	N/A	pCR rate: 100% (1/1); TRG G_0 rate: 100% (1/1)	None
Trojan, *et al.* (2021)[46]	Germany	Case series	1	$cT_3N_2M_0$	IHC + PCR	(Ipi + Nivo) × 1 cycle →TME	1	pCR rate: 100% (1/1)	None
Wang, *et al.* (2022)[47]	China	Retro	18[†]	$cT_3N_0M_0$	IHC ± PCR	Pembro/Sintili/Toripali/Camrelizu/Nivo ± Ipi (n = 3)/CAPOX (n = 3)/FOLFOXIR (n = 1)/apatinib (n = 1) → W&W	20.9	cCR rate: 100% (18/18); 2-yrs LRFS/DMFS/DFS/OS rate: 100% (18/18)	G3: Rash (n = 1); Hypoadrenalism (n = 1); Nervous system injury (n = 1)
Cercek, *et al.* (2022)[48]	USA	Pro, phase II	16	$cT_{3\text{-}4}N_0$ or $cT_xN_+M_0$	IHC	Dostarli × 9 cycles → W&W (cCR);	12	cCR rate: 100% (12/12)	None

						Concurrent CRT → TME (Non-cCR)			
Zhang, *et al.* (2022)[49]	China	Retro	8‡	$cT_{3-4}N_0$ or $cT_xN_+M_0$	IHC ± PCR	Pembro/Sintili/Tiselizu → W&W (cCR); TME ± adjuvant ICIs (Non-cCR)	14	cCR rate: 37.5% (3/8); ORR: 100% (8/8); PRR: 100% (8/8)	None
Bando, *et al.* (2022)[50]	Japan	Pro, phase II	5	$cT_{3-4}N_0$ or $cT_xN_+M_0$	N/A	CRT → Nivo × 5 cycles → TME	17.2	pCR rate: 60% (3/5); NAR score: 0.9 (0.9-20.4)	None
Hu, *et al*. (2022)[51]	China	Pro, phase II	4	$cT_{3-4}N_+M_0$	IHC ± PCR	Arm1: Toripal × 6 cycles → TME; Arm2: Toripali + Celecoxib × 6 cycles → TME;	N/A	Arm1: pCR rate: 50% (1/2); Arm2: pCR rate: 100% (2/2)	N/A
Kothari, *et al.* (2022)[52]	USA	Retro	1	$cT_4N_1M_0$	IHC	CT → Pembro × 16 cycles → TME	11	pCR rate: 100% (1/1)	N/A
Zhang, *et al.* (2022)[53]	China	Case series	1§	$cT_3N_2M_0$	IHC + PCR + NGS	SCRT → (CAPOX + Tislelizu) × 4 cycles → TME	11	pCR rate: 100% (1/1)	None
Mori, *et al.* (2022)[54]	Japan	Case series	1	$cT_4N_2M_0$	NGS	CAPOX × 4 cycles → CRT → Nivo × 4 cycles → TME	9	pCR rate: 100% (1/1)	None
Yang, *et al.* (2023)[55]	China	Case series	20	$cT_{3-4}N_0$ or $cT_xN_+M_0$	IHC/PCR/NGS	Arm 1: Tislelizu/Sintilib/Pembro × 6 (6-10) cycles → TME → ICIs × 2 (0-4) cycles; Arm 2: Tislelizu/Sintilib/Pembro × 8 (6-10) cycles → W&W	Total: 24.35; Arm 1: 24.5; Arm 2: 25	Total: ORR: 100% (20/20); pCR rate: 15% (3/20); cCR rate: 40% (8/20); 2-yrs LRFS/DMFS/OS: 100% (20/20); Arm 1: pCR rate: 84.6% (11/13); Arm 2: cCR rate: 42.9% (3/7); near cCR rate: 57.1% (4/7)	None
Chen, *et al.* (2023)[56]	China	Pro, phase II	16*	$cT_{3-4}N_0$ or $cT_xN_+M_0$	IHC ± PCR	Sintili × 4 cycles → a, TME → (Sintili ± CAPOX) × 4 cycles; b, Sintili → W&W (cCR); TME (Non-cCR)	17.2	cCR rate: 56.3% (9/16); pCR rate: 18.8% (3/16)	G3: Encephalitis (n = 1)
Li, *et al.* (2023)[57]	China	Retro	5**	$cT_3N_+M_0$	IHC/PCR/NGS	Pembro/Nivo/Sintili/Camrelizu ± CAPOX → W&W (cCR); TME (Non-cCR)	9.4	cCR rate: 40% (2/5); pCR rate: 40% (2/5)	N/A
Tissera, *et al.* (2023)[58]	Spain	Case series	1***	$cT_3N_2M_0$	IHC	XELOX × 3 cycles → PD → Pembro × 3 cycles → TME → Pembro	18.2	TRG G1 rate: 100% (1/1)	None
Pei, *et al.* (2023)[59]	China	Retro	1	$cT_3N_0M_0$	IHC + PCR/NGS	Sintili × 6 cycles → TME	11.2	pCR rate: 100% (1/1); DFS: 14.7 mths	None

Eefsen, *et al*. (2023)[60]	Denmark	Case series	1	$cT_3N_2M_0$	IHC	Pembro × 2 cycles → TME	26	2-yr LRFS/DMFS/DFS/OS rate: 100% (1/1)	None
Yu, *et al*. (2024)[61]	China	Retro	24	$cT_{3\text{-}4}N_0$ or $cT_xN_+M_0$	N/A	Anti-PD-1 Ab × 6 (1-12) cycles → W&W	34.5	cCR rate: 100% (24/24); 3-yr LRFS/DMFS/DFS/OS rate: 100% (24/24)	G3-4: Encephalitis (n = 1); Increased aminotransferase (n = 1)
Li, *et al*. (2024)[62]	China	Retro	3****	Patient 1: $cT_3N_1M_0$; Patient 2: $cT_3N_+M_0$; Patient 3: $cT_3N_+M_0$	IHC + PCR/NGS	Patient 1: CAPOX × 1 cycle → Pembro × 1 cycle; Patient 2: CAPOX × 1 cycle → Pembro × 2 cycles; Patient 3: CAPOX × 1 cycle → Sintili × 4 cycles → TME	9.7	Patient 1+2: cCR rate: 100% (2/2); Patient 3: TRG G3 rate: 100% (1/1)	N/A
Li, *et al*. (2024)[63]	China	Retro	2	Patient 1: $cT_{4b}N_{2a}M_0$; Patient 2: $cT_{4b}N_{2b}M_0$	PCR/NGS	Patient 1: Pembro × 1 cycle → TME; Patient 2: CAPOX × 2 cycles → Sintili × 6 cycles → TME	16	Patient 1: TRG G3 rate: 100% (1/1); Patient 2: pCR rate: 100% (2/2)	N/A
Tosi, *et al*. (2024)[64]	Italy	Retro	17	$cT_xN_{0/+}M_0$	IHC/PCR/NGS	Prior CT (n = 2)/RT (n = 1)/CRT (n = 1) → ICIs (Dostarli/Pembro/Nivo + ipi) × 4-6 cycles → W&W (n = 14)/TME (n = 3)	9.5	cCR rate: 94.1% (16/17); Near cCR rate: 5.8% (1/17) 1 pt developed unconfirmed lung metastases.	G3: Diarrhea (n = 1); Pneumonitis (n = 1)
Veselovsky, *et al*. (2024)[65]	Russian	Pro, phase II	2	Patient 1: $cT_4N_{2a}M_0$; Patient 2: $cT_{4a}N_{1b}M_0$	PCR ± IHC	Prolgoli → TME	N/A	pCR rate: 100% (2/2)	N/A

Abbreviations: dMMR = Defective mismatch repair system; LARC = Locally advanced rectal cancer; ICIs = Immune checkpoint inhibitors; CT = Chemotherapy; CRT = Chemoradiotherapy; IHC = Immunohistochemistry; PCR = Polymerase chain reaction; NGS = Next Generation Sequencing; ORR = Objective response rate; TRG = Tumor regression grade; cCR = Clinical complete response; pCR = Pathological complete response; pPR = Pathological partial response; MPR = Major pathological response rate; NAR = Neoadjuvant rectal; irAEs = Immune-related adverse effects; LRFS = Local recurrence-free survival; DMFS = Distant metastasis-free survival; DFS = Disease-free survival; OS = Overall survival; W&W, Watch and wait; N/A = Not available; CTCAE = Common Terminology Criteria for Adverse Events.

†Previous treatment regimens including FOLFOXIRI for 4 cycles (n = 1); XELOX for 5 cycles (n = 1); CRT and CT for 4 cycles (n = 1).

‡Two patients received ICIs after the failure of NCRT.

§The patient detected a liver lesion after receiving ICIs for 4 cycles and delivered radiofrequency ablation to the lesion.

*One patient discontinued after four cycles ICIs due to a serious adverse event.

**Two patients MMR and MSI status inconsistent: MMR status (IHC/NGS): pMMR, MSI status (PCR): MSS; MSI status (NGS): MSI-H.

***The patient detected a liver lesion after receiving XELOX for 3 cycles, and underwent excision of the segment V liver lesion after received ICIs for 3 cycles.

****The IHC and PCR/NGS results for Patient 3 are inconsistent, indicating pMMR by IHC and MSI-H by PCR/NGS.
